# The feasibility and efficacy of ultrasound-guided percutaneous laser ablation for multifocal papillary thyroid microcarcinoma

**DOI:** 10.3389/fendo.2022.921812

**Published:** 2022-08-17

**Authors:** Lu Zhang, Gui Ping Zhang, Wei Wei Zhan, Wei Zhou

**Affiliations:** Department of Ultrasound, Ruijin Hospital, School of Medicine, Shanghai JiaoTong University, Shanghai, China

**Keywords:** ultrasound guidance, thermal ablation, laser ablation, papillary thyroid microcarcinoma, multifocality

## Abstract

**Objective:**

The aim of this study was to evaluate the feasibility and efficacy of percutaneous laser ablation (PLA) for patients with multifocal papillary thyroid microcarcinoma (PTMC).

**Materials and methods:**

A cohort of patients who underwent ultrasound (US)-guided PLA for primary PTMC were enrolled in this study. The patients were divided into a multifocal PTMC (multi-PTMC) group and a unifocal PTMC (uni-PTMC) group. Before PLA, conventional US and contrast-enhanced ultrasound (CEUS) were performed to evaluate the PTMC and cervical lymph nodes. The operation time, energy, power, amount of isolation liquid, and complications during PLA were recorded. Patients were followed up at 2 days, 1 month, 3 months, and 6 months, and every 6 months after that. Volume reduction rate (VRR), local tumor recurrence, and lymph node metastasis after PLA were observed.

**Results:**

The study included 12 patients with 26 PTMCs and 60 patients with 60 PTMCs. The operation time, total energy, and amount of isolation liquid in the multi-PTMC group were more than those in the uni-PTMC group (*p* = 0.000, 0.007, and 0.020, respectively). The mean follow-up durations in multi-PTMC and uni-PTMC groups were 19.75 ± 11.46 months (6–36 months) and 16.33 ± 10.01 months (4–40 months), with a similar VRR of the ablated lesions in the two groups. One and three cases with newly developed PTMCs were observed in the multi-PTMC group and the uni-PTMC group during follow-up, respectively. There was no regrowth of treated lesions, lymph node metastasis, or distant metastasis. At the end of the follow-up, all the ablated lesions in the two groups completely disappeared or only remained scar strips.

**Conclusion:**

PLA is a safe and effective technique for treating multifocal PTMC, which might be an alternative technique for patients who are not eligible or are unwilling to undergo surgery.

## Background

Thyroid cancer is the most common malignant tumor among endocrine neoplasms, of which papillary thyroid microcarcinoma (PTMC) accounts for more than 84% (1). The traditional treatment strategy for PTMC is hemithyroidectomy or total thyroidectomy. However, the proportion of thyroid cancer in female cases attributable to overdiagnosis from 2008 to 2012 was about 87% in China (2). This indicates that there might be overtreatment of PTMC in clinical practice (3). The 2015 American Thyroid Association Management Guidelines for Adult Patients with Thyroid Nodules and Differentiated Thyroid Cancer suggested that an active surveillance approach can be considered in some patients with low-risk PTMC (4); however, it would evoke major anxiety if the tumor is left untreated, and repeated and regular ultrasound (US) examinations will be performed. Therefore, non-surgical thermal ablation treatments might be alternative methods for these cases.

Percutaneous laser ablation (PLA) is the first reported thermal ablation technology applied to PTMC (5). It can treat tumors by converting laser energy into thermal energy, which causes coagulation and necrosis. PLA is regarded as a minimally invasive treatment for solitary tumor due to its advantages of precise ablation range, high safety, minimal trauma, and quick postoperative recovery (6–10). However, previous studies mainly focused on the results of unifocal PTMC, rather than multifocal PTMC. The incidence of multifocality in papillary thyroid cancer (PTC) is 32%–39%, and it is often regarded as a risk factor, but the role in the prognosis remains unclear (11). The results of many studies on the role of multifocality in the clinical outcome of PTC were inconsistent and even contradictory (12–15). Several studies had been carried out on RFA for multifocal PTMC, and the results showed that RFA might be a promising treatment for both unifocal and multifocal PTMC (16, 17). To the best of our knowledge, few studies about PLA for multifocal PTMC have been reported. Therefore, this study was conducted to evaluate the feasibility and efficacy of PLA for patients with multifocal PTMC.

## Materials and methods

### Patients

This retrospective case–control study was approved by the ethics committee of our hospital. Twelve patients with 26 PTMCs and 60 patients with 60 PTMCs were treated with PLA in our hospital between June 2018 and September 2021. All the nodules were confirmed by fine-needle aspiration biopsy (FNAB). Relevant clinical and laboratory examinations were performed for all the patients before the PLA procedure, and written informed consent was obtained after informing patients of the possible risks and complications of treatment.

The inclusion criteria were as follows: (1) all the nodules were diagnosed as PTC by FNAB, with no sonographic evidence of capsular infiltration and extrathyroidal extension; (2) the maximum diameter of the nodule was no more than 10 mm; (3) in the multi-PTMC group, the number of PTMC in every patient was not less than two, and nodules were located in unilateral or bilateral lobes; and (4) patients were not eligible or were unwilling to undergo surgery. The exclusion criteria were as follows: (1) combination with other types of thyroid malignancies, such as medullary carcinoma, undifferentiated carcinoma, and thyroid metastatic carcinoma; (2) the nodule was located in the isthmus; and (3) detection of metastatic lymph nodes in the neck or distant metastasis.

### Preoperative evaluation

Medical history was obtained before PLA, and relevant examinations were performed, including thyroid function, liver and kidney function, blood routine and virus indicators analysis, chest x-ray/CT, and electronic laryngoscopy. US was performed using a real-time US instrument (Mylab Twice and Mylab 9, Esaote, Italy) equipped with multi-frequency linear probes (LA 523, L4-15 and LA 332, L3-11). The number, location, characteristics, volume of thyroid nodules, and bilateral cervical lymph nodes were carefully evaluated on imaging. Three radial lines were measured, including anteroposterior diameter, transverse diameter, and longitudinal diameter (V = πabc/6, V: volume, a: anteroposterior diameter, b: transverse diameter, and c: longitudinal diameter).

### PLA procedure

The patient laid on the operating table in a supine position with the neck extended. After local anesthesia was administered with 2% lidocaine, 0.9% normal saline was carefully injected into the space between the thyroid capsule and surrounding vital organs (common carotid artery, trachea, esophagus, and recurrent laryngeal nerve) to achieve fluid isolation. A 21G trocar was inserted into the tumor under US guidance. A 300-μm optical fiber was inserted into the trocar while retracting the needle by 5 mm, exposing the tip of the optical fiber to directly contact with the tumor. The treatment instrument was a high-power semiconductor laser that could continuously emit Nd-YAG laser at 1,064 nm (EchoLaser X4, Esaote, Florence, Italy). Ablation was suspended when the gasification and hypoechoic areas covered and completely exceeded the edge of the lesion by at least 2 mm, and the area of necrosis was assessed by contrast-enhanced ultrasound (CEUS) within 30 min to determine whether supplementary ablation was needed. At the end of the treatment, the laser was turned on while the trocar needle and optical fiber were withdrawn together to the outside of the thyroid envelope for ablating the needle tract. For multifocal PTMC, this procedure was first performed for one nodule and successively repeated for the other nodules.

### CEUS evaluation

Before CEUS, the background gain was adjusted to the echo of thyroid capsule just displayed, and the focus was placed on the deep surface of the target lesion. A bolus of 2.0–2.4 ml microbubble contrast agent (Sonovue, Bracco, Milan, Italy) was injected through the dorsal vein, followed by injecting 5 ml of normal saline. The timer was started immediately, lasting for 2 min. The whole process was recorded and stored on the US instrument’s hard drive. CEUS was performed to evaluate the perfusion intensity of every nodule before PLA and volume of perfusion defect after PLA.

### Therapy evaluation and follow-up

Complications, such as pain, bleeding, and nerve damage, were evaluated after PLA. The efficacy of local treatment was evaluated by conventional US and CEUS. Conventional US was performed at 30 min, 2 days, 1 month, 3 months, 6 months, and every 6 months after that. Conventional US mainly focused on the volume of the ablated area, tumor recurrence, and metastasis. CEUS was performed within 30 min and 2 days after PLA. On CEUS, the extent of non-enhanced area after ablation was evaluated to confirm that we had achieved a greater volume of necrosis than that of the nodule. We considered the complete ablation based on the following imaging findings: (1) conventional US showed the extent of the ablated area beyond the tumor border; and (2) CEUS showed the extent of the perfusion defect area significantly greater than the primary lesion. In cases which incomplete ablation was observed, a complementary ablation was performed immediately. Volume reduction rate (VRR) at different follow-up periods after PLA treatment was calculated. VRR = (V_30min_ − V_follow-up_)/V_30min_ (V_30min_ was the volume at 30 min after PLA, and V_follow-up_ was the volume at each time point of follow-up). US-guided FNAB was performed for lesions when the following items occurred during follow-up: (1) detection of insignificant reduction or regrowth of the ablated area; (2) detection of new suspicious nodules in thyroid during follow-up; and (3) detection of suspicious cervical lymph nodes.

### Analysis and statistics

Data were analyzed using the statistical software SPSS, version 19.0. Quantitative data that conformed to a normal distribution were described as mean ± standard deviation (SD), and compared by independent samples *t* test. Quantitative data that did not conform to a normal distribution were described as median (first quartile, third quartile), and compared by Mann–Whitney *U* test. Baseline characteristics of patients, preoperative sonographic features of conventional US and CEUS, and new lesions after PLA were compared with Fisher test. *p* < 0.05 was considered to indicate a significant difference.

## Results

### Baseline characteristics of participants

Between June 2018 and September 2021, we included 12 patients with multifocal PTMC and 60 patients with unifocal PTMC who were treated with PLA in our hospital. The baseline characteristics of patients and preoperative ultrasonic appearances of the PTMCs are listed in **Table 1**. In the multi-PTMC group, every patient had two to four nodules, with unilateral tumors in five cases and bilateral tumors in seven cases. The mean maximum diameter in the multi-PTMC group was smaller than that in the uni-PTMC group (*p* = 0.010), but the mean volume before PLA had no difference between the two groups (*p* = 0.059). The differences in the characteristics of conventional US and CEUS before PLA were not statistically significant (*p* > 0.05 for all).

**Table 1:** Characteristics of patients and PTMCs between the two groups before PLA.

Characteristics	Nodules/Patients, *n*/*N* (%)		*p*-value
	multi-PTMC	uni-PTMC	
Age	38.75 ± 6.80	39.33 ± 9.72	0.844
Sex			
Male	3/12 (25%)	11/60 (18.3%)	0.691
Female	9/12 (75%)	49/60 (81.7%)	
Maximum diameter (mm)	4.47 ± 1.48	5.38 ± 1.45	0.010
Volume (mm^3^)	28.61 (13.00, 64.07)	45.95 (31.13, 74.98)	0.059
Composition			
Solid	26/26 (100%)	59/60 (98.3%)	0.698
Solid-cystic	0/26 (0%)	1/60 (1.7%)	
Location			
Unilateral	5/12 (41.7%)	/	/
Bilateral	7/12 (58.3%)	/	
Calcification			
Microcalcification	6/26 (23.1%)	11/60 (18.3%)	0.515
Macrocalcification	1/26 (3.8%)	1/60 (1.7%)	
No calcification	19/26 (73.1%)	48/60 (80%)	
A/T (anteroposterior/transverse diameter ratio)			
A/T ≥ 1	15/26 (57.7%)	42/60 (70%)	0.299
A/T < 1	11/26 (42.3%)	18/60 (30%)	
Vascularity			
No vascularity	6/26 (23.1%)	8/60 (13.3%)	0.092
Low vascularity	20/26 (76.9%)	41/60 (68.3%)	
Moderate vascularity	0/26 (0%)	9/60 (15%)	
High vascularity	0/26 (0%)	2/60 (3.3%)	
CEUS intensity			
Low	5/26 (19.2%)	21/60 (35%)	0.211
Moderate	19/26 (73.1%)	37/60 (61.7%)	
High	2/26 (7.7%)	2/60 (2.3%)	

The treatment parameters during PLA, follow-up time, incidence of new PTMC, and lymph node metastases after PLA are listed in **Table 2**. There were statistically significant differences in the energy, operation time, and amount of isolation liquid between the two groups (*p* < 0.05 for all). The differences in the power, complications, incidence of new PTMC, and follow-up time were not statistically significant (*p* > 0.05 for all). The mean follow-up durations in multi-PTMC and uni-PTMC groups were 19.75 ± 11.46 months (range, 6–36 months) and 16.33 ± 10.01 months (range, 4–40 months), respectively.

**Table 2:** Characteristics during and after PLA between the two groups.

Characteristics of PLA	multi-PTMC (*N* = 12)	uni-PTMC (*N* = 60)	*p*-value
Complication			
No	12/12 (100%)	59/60 (98.3%)	1.000
Yes	0/12 (0%)	1/60 (1.7%)	
Power (W)	3.5 (3.5, 4.0)	4.0 (3.5, 4.0)	0.246
Energy (J)	889.35 ± 559.35	1,618.97 ± 864.72	0.000
Time (min)	26.28 ± 6.92	30.92 ± 6.77	0.007
Isolation liquid (ml)	18.62 ± 8.79	13.12 ± 10.24	0.020
New PTMC after PLA			
No	11/12 (91.7%)	57/60 (95%)	0.526
Yes	1/12 (8.3%)	3/60 (5%)	
Metastatic lymph node after PLA			
No	12/12 (100%)	60/60 (100%)	/
Yes	0/12	0/60	
Follow-up time (months)	19.75 ± 11.46	16.33 ± 10.01	0.352

All the patients tolerated the procedure well. In the uni-PTMC group, one patient developed a hematoma during ablation, which did not enlarge after 10 min of local compression, and the hematoma was absorbed within a week. There were no complications such as hoarseness, skin burns, esophageal injury, tracheal injury, or infection in both groups. The overall complication rates in the multi-PTMC and uni-PTMC groups were 0% and 1.7%, respectively, with no significant difference (*p* = 1.000). During follow-up, there were one and three new PTMCs in the multi-PTMC and uni-PTMC groups, respectively. One case in the multi-PTMC group was located in the right lobe, while two cases in the uni-PTMC group were located in the ipsilateral lobe and one in the contralateral lobe. A second PLA was performed for all the new PTMCs, and no more new malignancies were detected till the last follow-up. No regrowth of treated lesions, cervical lymph node metastasis, or distant metastasis was observed in either group.

### Comparison of treatment response in multi-PTMC and uni-PTMC groups

**Figure 1:**
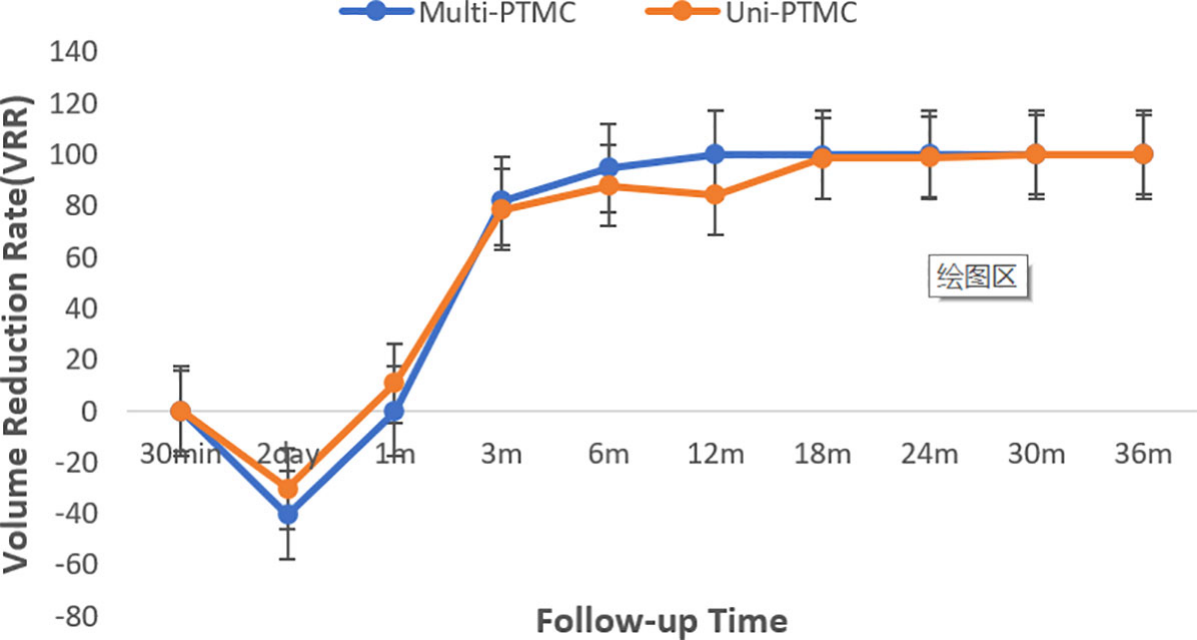
The diagram displayed the VRR at each follow-up after PLA.

All treatments were performed successfully as planned preoperatively. In both multi-PTMC and uni-PTMC groups, the mean volumes of the ablated lesions at 30 min after treatment were greater than those before treatment on CEUS. The absorption curve of the ablated lesions in the multi-PTMC group was coincident with that in the uni-PTMC group, which showed almost complete absorption at the end of follow-up (**Figure 1**). The mean volumes of the ablated lesions expanded at 2 days after PLA, with a gradual decrease at each subsequent follow-up visit in both groups.

**Figure 2:**
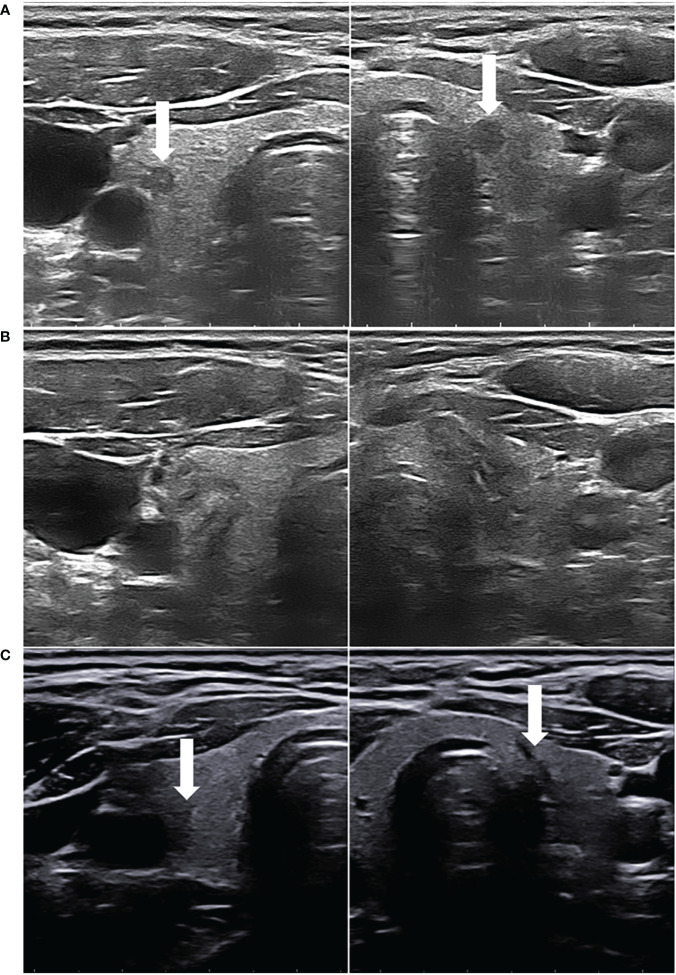
**(A)** A 33-year-old female patient had bilateral hypoechoic lesions (arrowhead), which were confirmed as PTMCs by FNAB. **(B)** After both the PTMCs underwent US-guided PLA, the ablated lesions appeared as well-defined heterogeneous echogenic areas on the grayscale US, with a central hypoechoic ablated needle channel. **(C)** On the final follow-up, the two ablated lesions were almost completely absorbed, leaving only scar strips (arrowhead).

**Table 3** shows the changes of mean volume and VRR at various follow-up periods between the two groups. The volumes of ablated lesions in the uni-PTMC group were significantly larger than those in the multi-PTMC group at 1 and 3 months of follow-up (*p* = 0.036 and 0.032). At 6 months after PLA, the VRR in the multi-PTMC group was smaller than that in the uni-PTMC group (*p* = 0.036). At the end of follow-up, all the ablated lesions completely disappeared or only remained scar strips (**Figure 2**). There were no statistical differences in volume and VRR at other time points of follow-up.

**Table 3:** Volume changes and VRR of the ablated lesions between the multi-PTMC group and the uni-PTMC group.

Time points of follow-up	Volume (mm^3^)		*p-* value	VRR (%)		*p-*value
	multi-PTMC	uni-PTMC		multi-PTMC	uni-PTMC	
30 min	335.79 ± 241.69	493.56 ± 297.1	0.340	/	/	/
2nd day	476.94 ± 295.71	689.35 ± 398.53	0.224	−40.42 ± 66.15	−30.22 ± 42.20	0.49
1st month	300.50 ± 229.57	439.84 ± 336.95	0.036	−0.10 ± 55.25	10.55 ± 47.80	0.401
3rd month	26.89 (10.27, 83.25)	62.50 (32.50, 126.00)	0.032	81.96 ± 15.90	78.52 ± 16.04	0.406
6th month	2.30 (0.00, 21.25)	25.50 (0.00, 56.75)	0.062	94.71 ± 8.14	87.91 ± 17.19	0.036
12th month	0.00	0.00 (0.00, 20.50)	0.074	99.05 ± 3.00	84.36 ± 23.74	0.316
18th month	0.00	0.00 (0.00, 0.75)	0.343	99.73 ± 0.85	98.43 ± 4.86	0.408
24th month	0.00	0.00	/	100	98.97 ± 3.51	0.447
30th month	0.00	0.00	/	100	100	/
36th month	0.00	0.00	/	100	100	/

## Discussion

The current guidelines and expert consensus on the thermal ablation of thyroid nodules rarely mentioned the indications for thyroid cancer. In 2018, Korean Radiological Society guidelines on radiofrequency ablation (RFA) of thyroid nodules suggested surgery as the standard method for primary thyroid cancer, and the indications for thermal ablation of primary thyroid cancer were not clearly defined (18). However, the safety, feasibility, and efficacy of thermal ablation for unifocal PTMC have already been reported (6–8, 19–24). Compared with conventional surgery, thermal ablation for PTMC has the advantages of less trauma, quicker recovery, and no scars. It can effectively shorten the hospitalization time and improve the quality of life of patients (25–29). Therefore, thermal ablation for PTMC was recommended in the guidelines of the Chinese Medical Doctor Association in 2020 and the European Thyroid Association and Cardiovascular and Interventional Radiological Society in 2021, but one of the inclusion criteria of PTMC must be unifocal (30, 31).

Tumor multifocality was suggested as an independent risk factor for the recurrence of PTC after total thyroidectomy, but the difference was significant only in those patients with PTC >1 cm but not in PTMC (11). In addition, multifocality was not an exclusion criterion in the active surveillance protocol for low-risk PTMC in the United States, Canada, and South Korea (32–34). Moreover, some studies with long-term follow-up revealed no significant differences in tumor size increase, lymph node metastasis, and extra-thyroidal invasion between unifocal and multifocal PTMC (35, 36). Although the current guidelines for the thermal ablation of PTMC did not recommend ablation of multifocal PTMC, several studies had been conducted on the clinical outcomes of RFA for multifocal PTMC (16, 17). A large cohort study evaluated the efficacy of RFA in 432 patients with unifocal PTMC and 55 patients with multifocal PTMC, and the results showed that multifocality had little effect on patient outcomes (16). According to the results in our study, the feasibility and efficacy of PLA for multifocal PTMC were preliminarily verified according to the short-term follow-up results.

Although both RFA and PLA can achieve good clinical outcomes for PTMC, many differences exist between the two techniques. First, heat-acting ends of PLA and RFA should be placed at the proximal and distal end of the nodule, respectively, before ablation, because the direction of heat propagation is mainly forward for PLA and backward for RFA. Second, in contrast to RFA, the temperature around optical fiber can reach over 200°C due to the absence of a cold circulation, allowing complete tumor inactivation through vaporization and carbonization. Third, compared to RFA, PLA results in a smaller area of necrosis due to limited lateral spread of heat, which might make it safer for nodules close to the recurrent laryngeal nerve or dangerous areas.

In 2011, the first case of PTMC treated by PLA was reported (5), and a 10-year follow-up study of PLA in the treatment of solitary PTMC was published in 2021 (37). In the past 10 years, many studies of PLA for unifocal PTMC have confirmed its high safety and efficacy. Valcavi et al. (38) treated three patients with unifocal PTMC by PLA followed by surgical removal of the thyroid gland. There was no thermal damage to the muscles, parathyroid gland, or the laryngeal recurrent nerve. The postoperative pathology showed tissue destruction and charring, with no tumor cell remnants, indicating complete inactivation. Short-term and long-term studies showed that PLA for unifocal PTMC had the advantages of precise ablation scope, high safety, low trauma, rapid postoperative recovery, and low recurrence rate (6, 8, 9, 37). There were no increases in complication rate, recurrence rate, and metastasis rate of PLA for unifocal PTMC compared with conventional surgery (26). However, the safety, clinical outcomes, and prognosis of PLA for multifocal PTMC have not been studied. In the present study, US-guided PLA was performed for unifocal PTMC and multifocal PTMC. Tumor reduction, new PTMC, and lymph node metastasis after treatment were compared between the two groups to analyze the feasibility and effectiveness of PLA in treating patients with multifocal PTMC.

In this study, both multifocal and unifocal PTMC were mainly presented as solid, non-calcified, and taller-than-wide nodules with low blood supply on conventional US and moderate perfusion intensity on CEUS before PLA. As the number of lesions increased, operation time, energy, and amount of isolation liquid significantly increased. In terms of complications, due to the increased number of punctures and prolonged operation time, the multi-PTMC group was expected to have a higher risk of complications such as bleeding; however, there was no difference in complications between the two groups.

The incidence of new PTMC and metastatic lesions after thermal ablation was about 0–5.6% (39). Bilateral multifocality was more aggressive than unilateral multifocality, but it was not an independent prognosis factor (40). In 2021, one study preliminarily evaluated the efficacy and safety of RFA for 47 patients with 100 bilateral PTMCs, and the incidences of lymph node metastasis and recurrence were 2.13% and 4.26%, respectively (17), which were not higher than the currently known incidences (37, 39). In this study, we included 7 (58.3%) patients with bilateral multifocal PTMCs. No lymph node metastasis was detected, and one patient (8.3%) developed a new PTMC. However, there were no significant differences in the incidences of new PTMC and lymph node metastasis between multi-PTMC and uni-PTMC groups over the similar follow-up period.

In our study, the volume of the necrotic area increased on postoperative day 2 in both groups, which might be attributed to the formation of microthrombi in the edematous area surrounding the necrotic site, inducing further tissue damage after PLA. The VRRs of the two groups gradually increased after 1 month until the ablated lesions almost disappeared, and the volume change curves were similar. Thus, the absorption rate in the multifocal PTMC group was not significantly different from that in the unifocal PTMC group according to the short-term follow-up. Although multifocal PTMC was not recommended as an indication for thermal ablation therapy according to the current guidelines and expert consensus, PLA is a potentially effective technique for multifocal PTMC according to our results. Surgery remains the first line of treatment for multifocal PTMC. However, with full informed consent, patients who refuse or are ineligible for surgery may choose PLA as an alternative treatment with strict follow-up.

Our study has some limitations. First, this was a retrospective study, and selection bias could not be avoided. Second, the sample size was small, and our results may not adequately represent differences in the safety and efficacy after PLA treatment between multifocal and unifocal PTMC. Third, the follow-up time was relatively short, and the long-term follow-up results were uncertain due to the indolent characteristic of PTMC.

In conclusion, short-term follow-up showed that safety and efficacy of PLA in the treatment of multifocal PTMC are similar to those of unifocal PTMC. It may be an alternative technique for patients who are not eligible or are unwilling to undergo surgery.

## Data availability statement

The original contributions presented in the study are included in the article/supplementary material. Further inquiries can be directed to the corresponding author.

## Ethics statement

This study was reviewed and approved by Ethics Committee of Ruijin Hospital, Shanghai Jiao Tong University School of Medicine. The patients/participants provided their written informed consent to participate in this study.

## Author contributions

LZ is the first author for performing PLA, data statistical and writing this manuscript. GPZ is a co-author for her arrangement of clinical data. WWZ is a co-author for his contribution in revision. WZ is the correspondence author for his organization, coordination and revision of this manuscript. All authors contributed to the article and approved the submitted version.

## Conflict of interest

The authors declare that the research was conducted in the absence of any commercial or financial relationships that could be construed as a potential conflict of interest.

## Publisher’s note

All claims expressed in this article are solely those of the authors and do not necessarily represent those of their affiliated organizations, or those of the publisher, the editors and the reviewers. Any product that may be evaluated in this article, or claim that may be made by its manufacturer, is not guaranteed or endorsed by the publisher.
